# Functional analysis of the involvement of apurinic/apyrimidinic endonuclease 1 in the resistance to melphalan in multiple myeloma

**DOI:** 10.1186/1471-2407-14-11

**Published:** 2014-01-08

**Authors:** Jiayin Xie, Liang Zhang, Mengxia Li, Jia Du, Liwei Zhou, Senlin Yang, Linli Zeng, Zengpeng Li, Ge Wang, Dong Wang

**Affiliations:** 1 Cancer Center, Research Institute of Surgery, Daping Hospital, Third Military Medical University, Chongqing 400042, P.R China; 2 Department of Pathology, Research Institute of Surgery, Daping Hospital, Third Military Medical University, Chongqing 400042, P.R China

**Keywords:** Acquired melphalan resistance, Multiple myeloma, Human apurinic/apyrimidinic endonuclease 1, DNA repair, Base excision repair

## Abstract

**Background:**

Melphalan resistance has been considered one of the major obstacles to improve outcomes in multiple myeloma (MM) therapy; unfortunately, the mechanistic details of this resistance remain unclear. Melphalan is a highly effective alkylating agent which causes many types of DNA lesions, including DNA base alkylation damage that is repaired by base excision repair (BER). We postulated that human apurinic/apyrimidinic endonuclease 1 (APE1), an essential BER enzyme, plays a vital role in acquired melphalan resistance. However, because APE1 is a multifunctional protein with redox activity and acetylation modification in addition to its major repair activity, the particular APE1 function that may play a more important role in melphalan resistance is unknown.

**Methods:**

Two MM cell lines, RPMI-8226 and U266 were used to measure the difference in APE1 levels in melphalan-resistant and sensitive derivatives. APE1 functional mutants for DNA repair, redox and acetylation were employed to investigate the roles of individual APE1 activities in acquired melphalan resistance.

**Results:**

Our results indicate that APE1 is overexpressed in both MM melphalan-resistant cells. Knocking down APE1 sensitizes the melphalan resistant MM cells to melphalan treatment. The exogenous expression of DNA repair mutant H309N and acetylation mutant K6R/K7R of APE1 failed to restore the melphalan resistance of the APE1 knockdown RPMI-8226 cells. The AP endonuclease activity and multidrug resistance protein 1 (MDR1) regulatory activity may play roles in the melphalan resistance of MM cells.

**Conclusions:**

The present study has identified that the DNA repair functions and the acetylation modification of APE1 are involved in melphalan resistance of MM cells and has also shed light on future therapeutic strategies targeting specific APE1 functions by small molecule inhibitors.

## Background

Primary drug resistance in multiple myeloma (MM) patients has created a hurdle to consistently successful chemotherapeutic outcomes. Despite gradual advances in treatment using optimized strategies that combine multiple agents, effective remission is achieved in a sub-optimal number of patients (fewer than 50% of patients) [1]. The current standard of care for elderly MM patients includes the nitrogen mustard alkylating agent melphalan in conjunction with prednisone. Melphalan primarily distorts the DNA guanine base with an alkyl group monoadduct [2], particularly at the nitrogen atom 7 of the imidazole ring, and it can also distort DNA with other adducts. Several suspected means of in vitro resistance to these mustard agents include cytokine production defects in the bone marrow milieu, altered drug delivery by transporters that effectively decrease cellular drug absorption, and an increase in effective DNA repair of mustard-specific lesions [3,4]. Currently it is unclear which of these pathways contributes to drug resistance during chemotherapeutic regimens.

Among all the previously-proposed mechanistic models, enhanced DNA repair capacity has been closely associated to melphalan resistance in MM patients [5,6]. DNA repair function has been widely accepted as the most important mechanism of resistance to anticancer drugs, especially those specifically targeting DNA. Although there are 5–6 major DNA repair pathways, the major type of DNA damage caused by melphalan is base alkylation which is mainly repaired by DNA base excision repair (BER) mechanisms [7]. Melphalan-induced alkylated bases are recognized and removed by particular glycosylases such as methylpurine glycosylase (MPG) leaving an abasic site with the N-glycosyl bond intact. Then human apurinic/apyrimidinic endonuclease 1 (APE1) cleaves the DNA backbone at the abasic site, leaving an exposed 3′OH and 5′ deoxyribose phosphate. Following the function of APE1, DNA polymerase beta incorporates the correct nucleotide followed by nick ligation by ligase III. In the BER pathway, the activity of APE1 largely determines the effectiveness of this DNA repair [8]. APE1 is an essential protein for many cellular processes [9] and its biological importance is highlighted by early embryonic lethality in mouse Apex1, the homologue of human APE1, knockout mice [10]. A number of preclinical functional studies revealed that APE1 is more highly expressed in various types of tumor tissues which supposedly contributes to cancer cell survival and proliferation [11,12]. Moreover, overexpression of APE1 in tumor tissues is usually closely correlated with a less effective response or resistance to cancer therapeutic agents [13,14]. Although the role of APE1 in drug resistance has been established and widely accepted, the detailed mechanisms for individual therapeutic agents may vary and are not yet fully understood.

We previously reported that APE1 is involved in melphalan resistance in the MM cell line KM3 by using a tissue-specific inducible APE1 silencing vector [15]. As a result, we confirmed that APE1 is a promising therapeutic target and that suppressing the expression of APE1 might enhance melphalan treatment in MM patients. However, APE1 is a multifunctional protein with at least two distinct activities which play different roles in drug resistance. As mentioned above, APE1 is the essential enzyme of DNA base excision repair [16]. On the other hand, APE1 is a redox factor regulating important agents involved in oxidative stress, including NF-κB, AP-1, p53, and Egr-1 [17]. Notably, recent studies indicate a novel role of APE1 in regulating MDR1 expression through an acetylation-dependent mechanism [18]. The membrane-associated protein encoded by the Mdr-1 gene is a member of the superfamily of ATP-binding cassette (ABC) transporters which functions as an ATP-dependent drug efflux pump for xenobiotic compounds [19]. Therefore, it is responsible for decreased drug accumulation in multidrug-resistant cells which further facilitates the development of drug resistance.

Taken together, overexpression of APE1 in MM cells promotes resistance to melphalan, possibly through different mechanisms, while the involvement of different activities of APE1 in this process remains unknown. Therefore, we initiated the present study to explore which APE1 functions are involved in melphalan resistance in MM cells. We utilized APE1 overexpression or RNAi vectors to measure the impact of manipulating APE1 on melphalan resistance of the MM cell lines. Additionally, we used APE1 function-specific or post-transcriptional modification site mutant overexpression vectors to differentiate the impact of specific APE1 activities on melphalan resistance. Our results indicated that APE1 overexpression and manipulation in melphalan resistant MM cells affects melphalan resistance. The DNA repair activity and MDR1 regulatory activity of APE1 are mainly involved in the melphalan resistance of RPMI-8226 MM cells while DNA repair activity is more important in cell survival following melphalan treatment.

## Methods

### Cell and reagents

RPMI 1640 medium, Opti-MEM® I Reduced-Serum Medium and fetal bovine serum, Lipofectamine™ 2000 Transfection Reagent, TRIzol RNA isolation reagent and primers were from Invitrogen (Grand Island, NY). Cell Counting Kit-8 (CCK-8), melphalan, myrecitin, synthetic siRNA against APE1 and MDR1 were from Sigma-Aldrich (St Louis, MO). Tetrahydrofuran-containing oligonucleotides, biotin-conjugated oligos and all other regular oligos were synthesized from Takara (Dalian, China). T4 polynucleotide kinase (T4 PNK), T4 ligase, restriction endonucleases, and high-fidelity Pfu DNA polymerase were from Promega (Madison, WI). Halt protease inhibitor cocktail, LightShift chemiluminescent EMSA kit, Super Signal West Pico chemiluminescent reagents, horseradish peroxidase-conjugated anti-mouse or anti-rabbit IgG antibodies were from Pierce (Rockford, IL). The chemo-sensitive RPMI-8226 and U266 cell line were purchased from American Type Culture Collection (Manassas, VA) and their melphalan-resistant sublines RPMI-8226/LR5 and U266/LR6 were obtained from Dr. William S. Dalton (Lee Moffitt Cancer Center, Tampa, FL). All MM cell lines were grown in RPMI 1640 medium supplemented with 10% fetal bovine serum, 100 U/ml penicillin and 100 μg/ml streptomycin (Hyclone, Logan, UT), and maintained at 37°C in a humidified atmosphere in the presence of 5% CO_2_-95% air.

### Constructs and transfection

The constructs of APE1 knockdown and wildtype or overexpression mutants used in this study included pTer/APE1 (shAPE1), p3XFLAG-CMV/APE1^WT^ (APE1^WT^), p3XFLAG-CMV/APE1^H309N^ (APE1^H309N^), p3XFLAG-CMV/APE1^C65S^ (APE1^C65S^), and p3XFLAG-CMV/APE1^K6R/K7R^ (APE1 ^K6R/K7R^), kind gifts from Dr. Gianluca Tell (University of Udine, Udine, Italy). The APE1 eukaryotic overexpression vector was constructed based on the pcDNA-3.1 vector. The detailed procedures were reported previously [9,20]. The transfections were performed using Lipofectamine™ 2000 Transfection Reagent following the manufacturer’s protocol for transient transfection of suspension cells.

### CCK-8 assay

Cells (2 × 10^5^) on 6-well plates were transfected or treated as indicated. Cell viability was evaluated by MTT assay at various time points after transfection or treatment. Cell-counting kit-8 reagent was added to each dish at a concentration of 1/10 volume, and the plates were incubated at 37°C for an additional 4 h. Absorbance was then measured at 490 nm and at 630 nm as a reference with a Microplate Reader 550 (Bio-Rad Laboratories, Hercules, CA). Cell viability (%) = OD value of treatment group/OD value of control group × 100%.

### Western blot and antibodies

Western blots were performed as previously described [21]. Suppliers and incubation conditions of antibodies used for Western blots were as follows: anti-APE1 monoclonal (Novus), 1 h at 37°C, dilution 1:5000; anti-MDR1 monoclonal (Sigma), dilution 1:500; HRP-conjugated anti-acetylation lysine antibody (Milipore), dilution 1:1000, overnight at 4°C; anti-β-actin monoclonal (Sigma), 1 h at 37°C, dilution 1:2000.

### Quantitative RT-PCR

Expression of the APE1 gene was detected by real-time RT-PCR and normalized by control gene β-actin expression. Total RNA was extracted using the TRIZOL reagent and then reverse-transcribed into single-stranded DNA using PrimeScript™ 1st Strand cDNA Synthesis Kit (Takara, Dalian, China). Real-time RT-PCR was performed with a Lightcycler 480 real-time RT-PCR system (Roche Diagnostics). APE1 forward primer, 5′-TGGAATGTGGATGGGCTTCGAGCC-3′ and APE1 reverse primer, 5′-AAGGAGCTGACCAGTATTGATGA-3′ were utilized.

### Oligonucleotide cleavage assay

The AP endonuclease activity of APE1 was evaluated by a well-characterized oligonucleotide cleavage assay [21]. Briefly, a 51-mer oligonucleotide containing a THF site, the analogue of an abasic site, at the 22nd position was 5′-end radiolabeled. The labeling reaction consisted of 10 pmol of the single stranded oligonucleotide, 2.5 pmol of γ^32^P-ATP, T4 PNK, and appropriate kinase buffer in a total volume of 10 μl. Reactants were incubated for 30 minutes at 37°C and 5 minutes at 95°C. Complementary oligonucleotide was then added and cooled down to 22°C to form duplex DNA. Activity assays contained 0.5 pmol of labeled duplex oligonucleotide, 1 × REC Buffer [50 mM HEPES, 50 mM KCl, 10 mM MgCl_2_, 1% (w/v) bovine serum albumin, 0.05% (v/v) Triton X-100 (pH 7.5)], protein extraction (0 to 10 μg) in a 10 μl reaction volume and were incubated at 37°C for 15minutes. The reactions were terminated by adding 10 μl formamide with dyes. Equal volumes (20 μL) of the reaction products from assays of AP endonuclease activity were resolved in a 20% polyacrylamide gel with 7 M urea in 1 × Tris-borate EDTA buffer at 300 V for 40 minutes. Wet gels were autoradiographed at −70°C overnight.

### Electrophoretic mobility shift assay (EMSA)

EMSA was performed according to the LightShift Chemiluminescent EMSA kit user’s instructions with minor modifications. Briefly, 5 μg of nuclear extracts were incubated with 3′-biotin labeled, purified double-stranded oligonucleotide probes. The probes containing NF-κB consensus sites: NF-κBF: 5′-AGTTGAGGGGACTTTCCCAGGC-3′ and NF-κBR: 5′- CGGACCCTTTCAGGGGAGTTGA -3'were synthesized and labeled with biotin at the 3′ end. After incubation for 30 min at room temperature, samples were separated on a pre-run 5% polyacrylamide gel at 100 V for 90 min and then transferred to Zeta-Probe GT nylon membrane (Bio-Rad). The probes were detected by HRP-conjugated streptavidin (1:300) and the bands were visualized by ECL reagents provided with the kit. The resultant bands were quantified using the imaging software Quantity One (Bio-Rad).

### Comet assay

The cells were rinsed twice with ice-cold PBS and harvested, and the cell suspension was exposed to melphalan on ice for 15 min. Immediately after treatment or after a 30 min recovery incubation at 37°C post treatment, the cell suspension was stored on ice and an alkaline comet assay performed using a Comet assay kit (Trevigen, USA) according to the manufacturer’s instructions with modifications.

### Co-immunoprecipitation assay

Cells were harvested by scraping and washed once with ice-cold phosphate-buffered saline (PBS). Cell pellets were resuspended and incubated in IP lysis buffer (Beyotime Institute of Biotechnology, Jiangsu, China) supplemented with protease inhibitor cocktail (Pierce) at a cell density of 10^7^ cells/ml on ice for 30 min. After centrifugation at 12,000 × g for 10 min at 4°C, the supernatant was collected as total cell lysate. Protein concentration was determined by using the Bradford assay (Bio-Rad, Hercules, CA). Samples were pre-cleared by incubating with protein A/G agarose resin for 30 min on ice then coimmunoprecipitated for 3 h using APE1 antibody (Novus) following the manufacturer’s instructions. After incubation, protein A/G agarose resin was then added and incubated for 1 hour at 4°C. After washing 3 times with PBS containing protease inhibitor, the pellet containing agarose beads together with binding proteins were mixed with sample buffer and incubated at 100°C for 5 min. The samples were then stored at −80°C or subjected to Western blotting analysis immediately.

## Results

### The differential expression of APE1 in RPMI-8226 and U266 parental cell and their drug-resistant cell lines RPMI-8226/LR5 and U266/LR6

**Figure 1:**
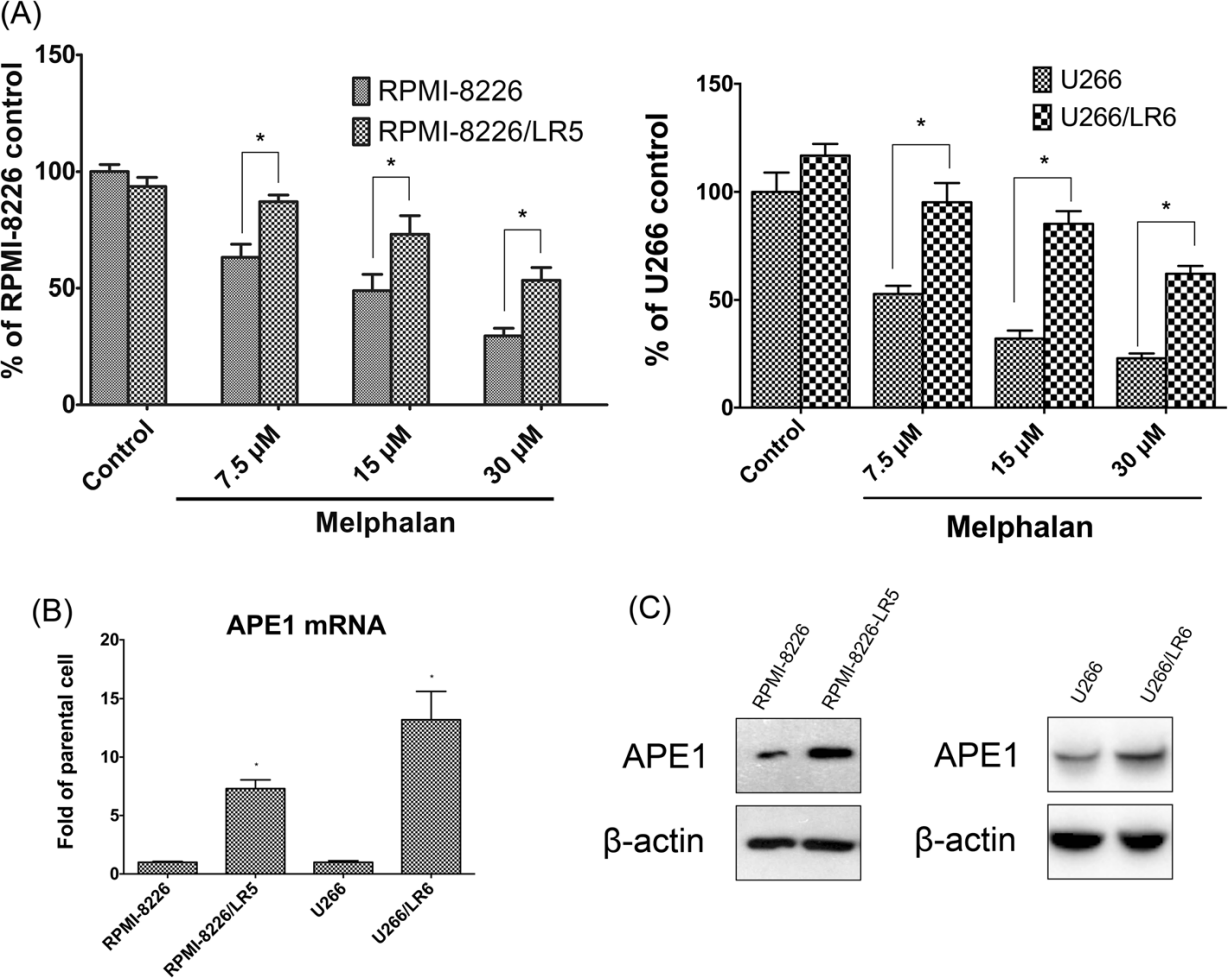
**APE1 overexpression in melphalan-resistant MM cell lines RPMI-8226/LR5 and U266/LR6. (A)** CCK-8 assay indicated that RPMI-8226/LR5 and U266/LR6 show more resistance to melphalan than their parental cell lines RPMI-8226 and U266. All cell lines were treated with three different doses of melphalan for 48 hours then cell viability was assayed using the CCK-8 assay. Results are shown as mean ± SD and were from three separate experiments. The significance was analyzed by Student *t* test. Stars (*) represent that the differences between RPMI-8226/LR5 and RPMI-8226 or between U266/LR6 and U266 are statistically significant (*p* < 0.01). Differential APE1 expression at the mRNA **(B)** and protein **(C)** level was assayed using quantitative RT-PCR and Western blots, respectively. The bar graph showing the quantitative results of APE1 mRNA levels was from three independent experiments. Representative blot images are shown and β-actin was used as a loading control.

**Figure 2:**
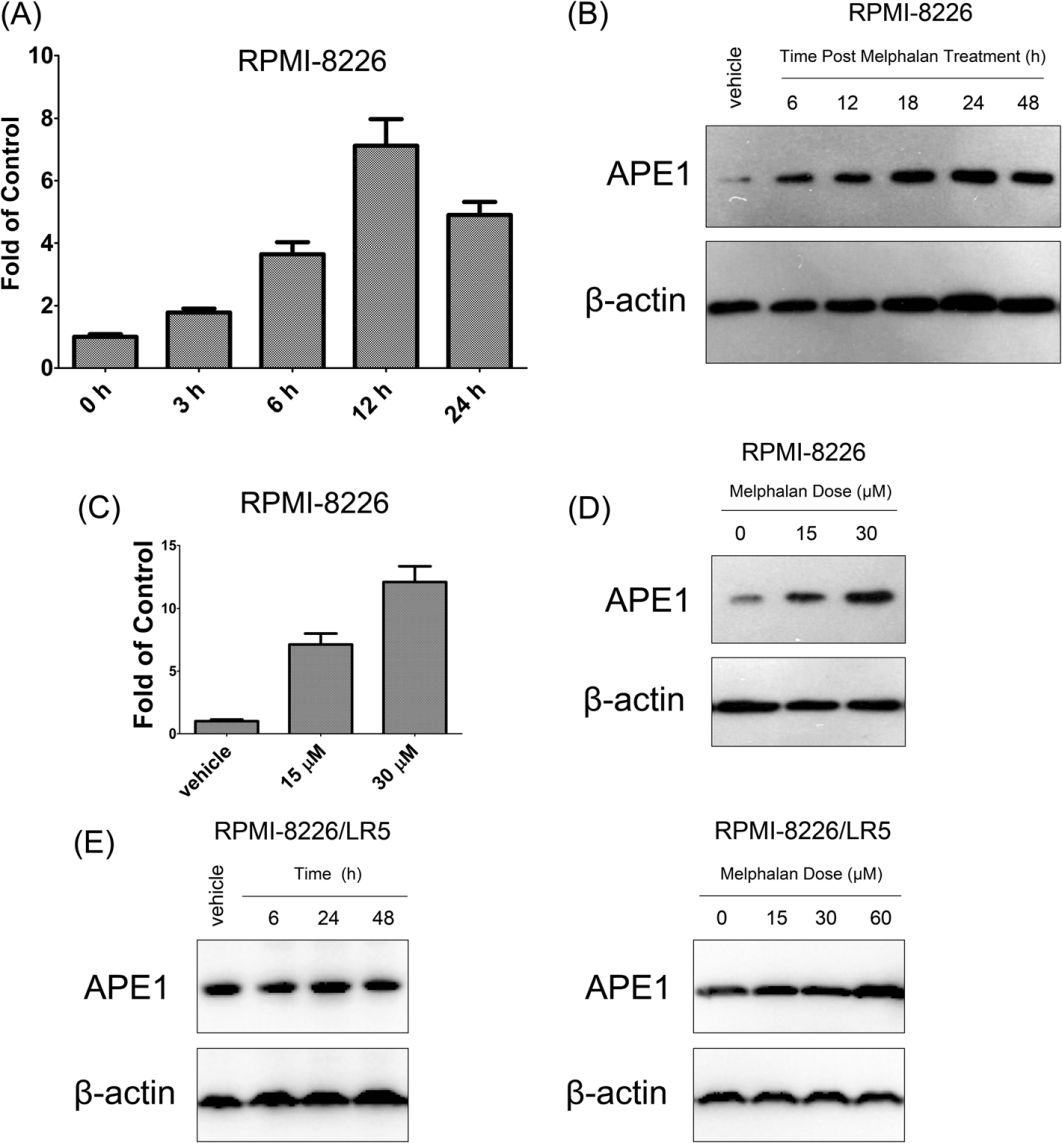
**APE1 responds to melphalan treatment.** Both mRNA and protein levels were elevated after melphalan treatment in a time course in RPMI-8226 cells. Quantitative PCR results are shown as a bar graph in **(A)** and representative Western blot images are shown in **(B)**. In addition, expression of APE1 mRNA **(C)** and protein **(D)** levels increased in a melphalan dose dependent manner. **(E)** APE1 protein level alterations in melphalan-resistant line RPMI-8226/LR5 were tested by Western blot at different time points post 15 μM melphalan treatment (left panel) or at 24 hours post various doses of melphalan treatment (right panel). All quantitative RT-PCR results were statistically processed from three independent experiments, and the blot is a representative of three independent Western blots. Stars (*) represent that the difference between the indicated group and DMSO (vehicle) treated RPMI-8226 is statistically significant (*p* < 0.01).

To investigate the role of APE1 in melphalan resistance of multiple myeloma cells, we utilized melphalan-resistant MM cell lines RPMI-8226/LR5 and U266/LR6 and their parental cells. The drug resistant statuses of all cell lines were validated by CCK-8 assay (Figure 1A). The results suggested that the proliferation of 8226/LR5 and U266/LR6 cells are slightly inhibited by different doses of melphalan while the growth of the parental cell lines are significantly suppressed by the drug (all *p* values < 0.01). The differential expression of APE1 was then measured for both mRNA and protein levels in these two groups of cell lines by quantitative RT-PCR and Western blot, respectively. Figure 1B and C show both APE1 mRNA and protein levels were higher in the melphalan-resistant cell lines (both *p* values <0.01) suggesting that APE1 is a melphalan responsive gene. To check this hypothesis we challenged the cells with 15 μM melphalan for 1 hour then measured APE1 expression differences in both wildtype RPMI-8226 and RPMI-8226/LR5 cells. As shown in Figure 2A and B, APE1 mRNA expression was elevated after melphalan treatment as early as 3 hours while protein level was elevated at 18 hours after treatment. The peak of APE1 protein elevation was at 24 hours after melphalan treatment and the mRNA peak was at 12 hours. The significant elevation of APE1 expression was observed in a dose dependent fashion at 24 hours post melphalan treatment (Figure 2C and D). On the other hand, the APE1 level, which is already high in RPMI-8226/LR5 cells, failed to show a significant increase until high dose treatment of melphalan (60 μM). These correlative data suggested that APE1 could play a role in a melphalan-induced cellular response and consequently promote resistance to melphalan.

### Manipulation of APE1 affects cell resistance to melphalan

**Figure 3:**
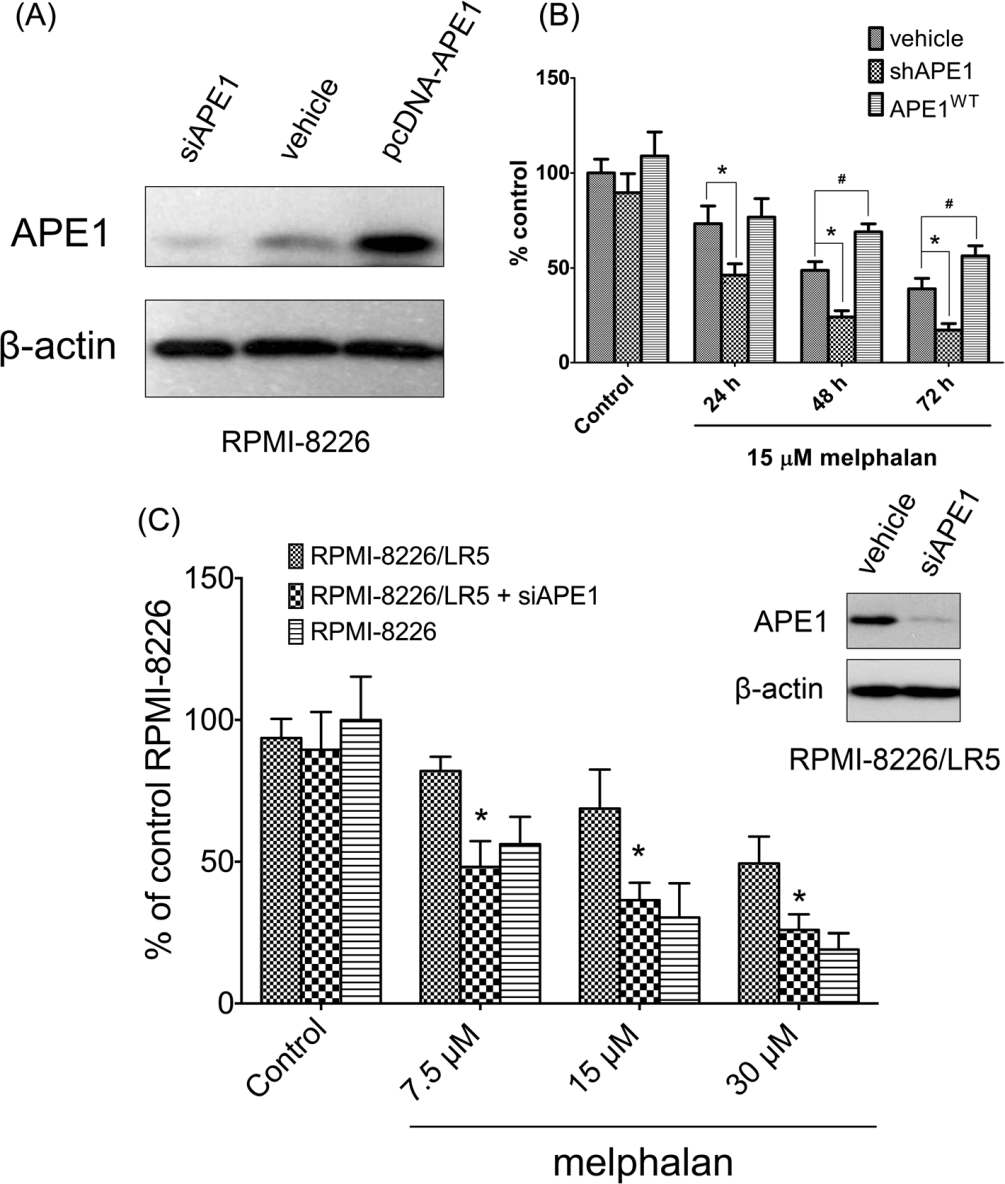
**Manipulation of APE1 affects cell resistance to melphalan. (A)** APE1 protein levels at 48 hours post transfection of siRNA or overexpression vector in RPMI-8226 cells were measured using Western blot. Transfection reagent only treated cells were included as a control. **(B)** Cell survival after 24, 48 and 72 hours post 15 μM melphalan treatment in all three groups was measured using CCK-8 assay. A star (*) represents that the difference between the shAPE1 transfected group and the vehicle alone group is statistically significant (*p* < 0.05), # represents that the difference between pcDNA-wtAPE1 transfected group and vehicle alone group is statistically significant (*p* < 0.05). **(C)** APE1 knockdown in RPMI-8226/LR5 partially restored the sensitivity to melphalan. The results shown in the bar graph indicated the cell viability in different groups after melphalan treatment using the CCK-8 assay. The representative Western blots show APE1 was effectively knocked down in RPMI-8226/LR5 by siRNA transfection. A star (*) represents that the difference between siAPE1 and vehicle alone transfected RPMI-8226/LR5 is statistically significant (*p* < 0.05), # represents that the difference between shAPE1 transfected RPMI-8226/LR5 and vehicle alone transfected RPMI-8226 is statistically significant (*p* < 0.05). All results were from three independent experiments.

To further confirm the role of APE1 in melphalan resistance, we utilized RNAi and vector-based overexpression strategies to manipulate cellular APE1 expression in wildtype RPMI-8226 cells. The changes in drug resistance were then observed in cells with exogenously altered APE1 expression. RNAi was performed using adenovirus previously engineered by our lab and its efficacy was confirmed by Western blot [22]. Both RNAi and overexpression effectively altered total cellular APE1 protein levels at 48 hours after transduction according to the Western blot shown in Figure 3A. Noteworthy, since the B cell origin of both parental MM cell lines, we found that the APE1 knockdown rendered no significant growth inhibition under untreated conditions, which agrees with previous reports [23,24]. The melphalan resistance was then tested by CCK-8 assay in the groups with different APE1 expression levels. The cell killing effects by melphalan were measured at 24, 48 and 72 hours after 15 μM melphalan treatment. Figure 3B clearly indicates that APE1 deficiency sensitized RPMI-8226 cells to melphalan; meanwhile, overexpression of APE1 rendered 8226 cells with enhanced resistance to melphalan. In addition, we also tested if APE1 inhibition in RPMI-8226/LR5 cells could restore the sensitivity to melphalan. At 48 hours post transfection, APE1 protein levels were effectively downregulated as shown in Figure 3C. At 48 hours following melphalan treatment, CCK-8 assays were performed to measure the cell viability in different groups. The results indicated that APE1 knockdown significantly decreased RPMI-8226/LR5 survival under various doses of melphalan treatment (*p* < 0.01), and it restored the melphalan sensitivity of RPMI-8226/LR5 to the level of its parental cell line (*p* > 0.05).

### DNA repair is involved in melphalan-resistant MM cells

**Figure 4:**
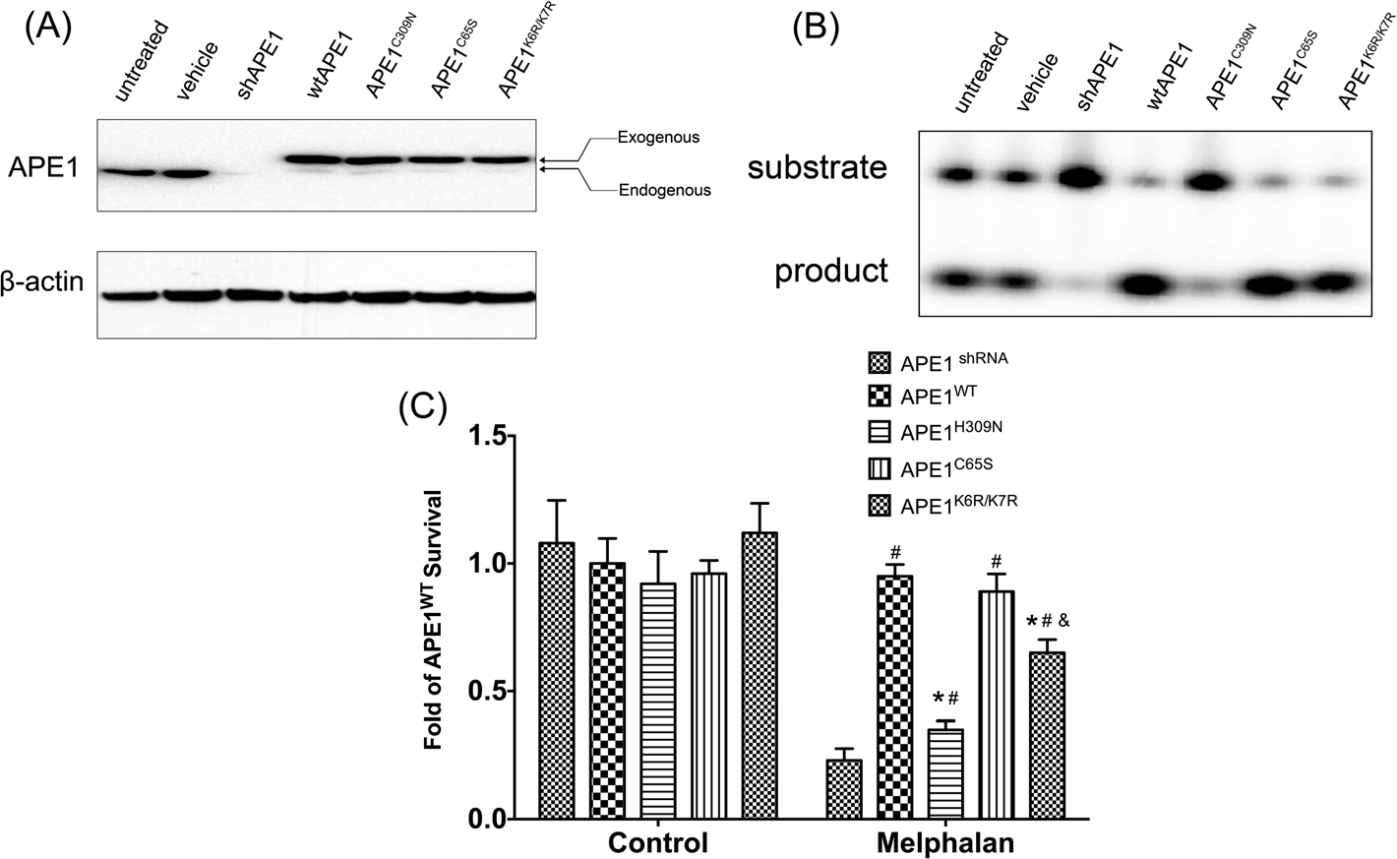
**Differential involvement of various APE1 functions in melphalan-resistanct MM cells. (A)** The protein expression levels of APE1 mutants were assayed by Western blot and the results indicated that the exogenous protein levels were comparable in APE1 ^WT^, APE1^H309N^, APE1^C65S^ and APE1^K6R/K7R^ cell lines. **(B)** The AP endonuclease activities of the whole cell extracts from APE1^shRNA^ and exogenous APE1-expressing cells were measured by oligo incision assay. The representative image out of three experimental repeats was shown. **(C)** Cell survival of different APE1 mutant-transfected groups was measured by CCK-8 assay after various doses of melphalan treatment. All results were from three independent experiments and * represents that the difference between the indicated group and the APE1 ^WT^ group is statistically significant (*p* < 0.01), # represents that the difference between the indicated group and APE1^shRNA^ group is statistically significant (*p* < 0.01), and & represents that the difference between the indicated group and the APE1 ^H309^ group is statistically significant (*p* < 0.01).

To comparatively investigate the importance of different functions of APE1 in melphalan resistance of RPMI-8226, three constructs expressing loss-of-function mutants of APE1 were introduced. Since APE1 is generally an abundantly expressed protein, we first knocked down its expression by shAPE1 adenovirus. As shown in a previous study, the APE1 expression level was suppressed for more than 96 hours which gave us a window to further manipulate the APE1 expression and measure the biological changes. APE1^H309N^, APE1^C65S^ and APE1^K6R/K7R^ represent repair activity deficiency, redox activity deficiency, and acetylation site mutants that were separately transfected into APE1 at 24 hours post shAPE1 adenovirus infection in RPMI-8226/LR5 cells. At 24 hours post transfection, the overall APE1 expression was measured by Western blot using APE1 antibody. As shown in Figure 4A, the expression of the three mutants together with the wildtype APE1 control (APE1^WT^) was basically the same at 24 hours post transfection. Additionally, the exogenously expressed APE1 or its mutants demonstrated the same expression level or even more than the endogenous APE1 which resulted in significant biological changes by the exogenous mutants. The AP endonuclease activities of different mutants were tested by oligo incision assay. As shown in Figure 4B, when normalized to the APE1 protein level, the H309N mutant demonstrated significant loss of AP endonuclease activity of APE1, whereas other mutants demonstrated similar activity to the wildtype cell line. The sensitivities to melphalan of different groups were then measured by CCK-8 assay and the results indicated that the knockdown of APE1 sensitized the RPMI-8226/LR5 cell to melphalan while APE1^WT^ transfection restored the resistance (Figure 4C). In melphalan untreated groups, the expression of different APE1 mutants rendered the same cell survival as wildtype APE1 expression at the time of 72 h (*p* > 0.05). When transfected with APE1^H309N^, APE1^C65S^ and APE1^K6R/K7R^, the melphalan resistance of APE1 knockdown cells was partially restored to different levels. APE1^H309N^-transfected cells with DNA repair activity deficiency were significantly more sensitive to melphalan compared to APE1^WT^ using the student *t* test (*p* < 0.01). Meanwhile, APE1^C65S^ restored melphalan resistance as much as APE1^WT^ without statistical significance (*p* > 0.05) and APE1^K6R/K7R^ restored resistance to a level between APE1^C65S^ and APE1^H309N^, but significantly lower than the APE1^WT^ group (*p* < 0.05). These results demonstrated that both DNA repair activity and acetylation modification of APE1 participate in regulating cell survival after melphalan treatment.

### The DNA repair activity of APE1 plays an important role in melphalan resistance

**Figure 5:**
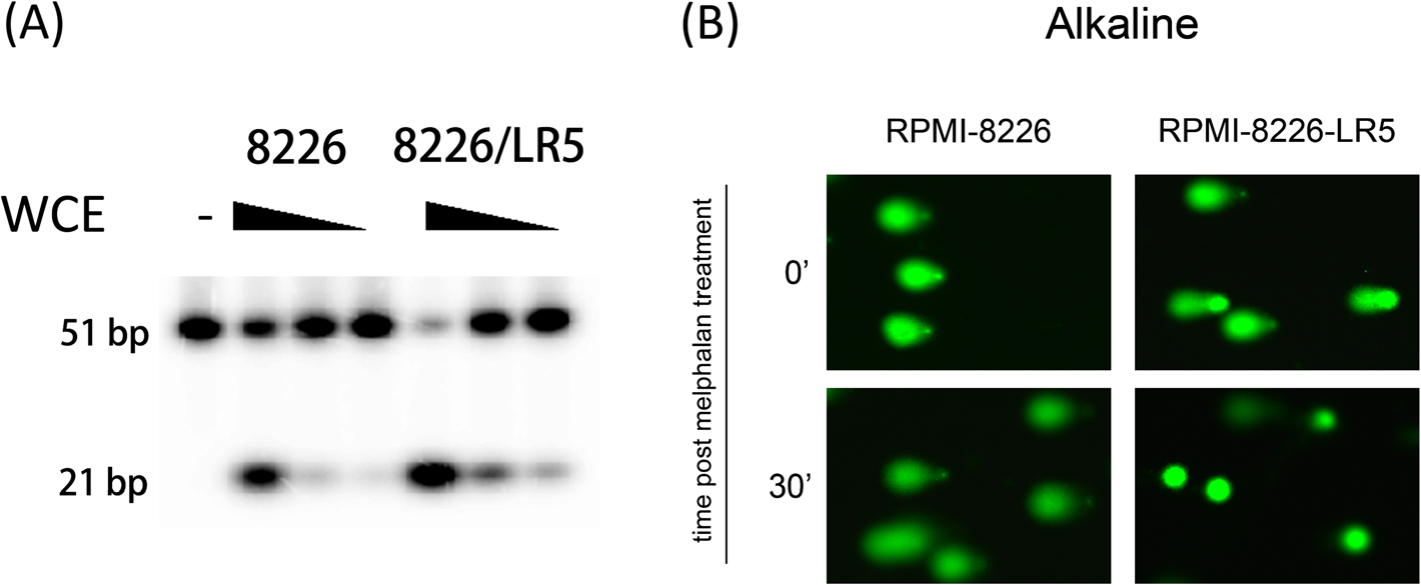
**DNA repair activity of APE1 plays a critical role in melphalan resistance of MM cells. (A)** The difference in DNA repair activity of APE1 between RPMI-8226/LR5 and RPMI-8226 cells was analyzed using AP site incision assay. The results indicated that the RPMI-8226/LR5 cells possess a higher AP endonuclease activity than its parental line. **(B)** The overall DNA repair activity for single DNA strand breaks was assayed by the alkaline comet assay. Cell suspensions from both RPMI-8226/LR5 and RPMI-8226 cells were treated with 15 μM melphalan on ice for 20 min, then the comet assay was performed immediately or after a 30 min repair in culture medium in a 37°C incubator. Representative images are shown.

To further explore the mechanism of the multiple APE1 activities in melphalan resistance, the DNA repair function of APE1 was analyzed first. When we tested the AP endonuclease by abasic site-containing oligonucleotide incision assay, the APE activity was significantly higher (2.19 ± 0.187 fold, *p* < 0.01) in RPMI-8226/LR5 cells compared to parental cells (Figure 5A). The results indicated that the repair activity of APE1 probably participates in the melphalan resistance but is also due to elevated expression. To verify that the DNA lesions caused by melphalan were less prominent in melphalan resistant cells, an alkaline comet assay reflecting base or small patch DNA lesions was performed. In congruence with APE activity, DNA lesions induced by melphalan were repaired effectively in RPMI-8226/LR5 compared to the parental cells which partially explained the differential sensitivity to melphalan. The reduced DNA repair capacity of APE1 rendered more DNA single strand breaks in RPMI-8226 at 30 min post melphalan treatment when compared to RPMI-8226/LR5 (Figure 5B). The overall DNA repair capacity of single stand breaks induced by melphalan further confirmed that the DNA repair activity of APE1 plays a critical role in melphalan resistance. Taken together, our results indicated that APE1 DNA repair activity plays an important role in melphalan resistance. However, the DNA repair function mutant still increases the melphalan resistance in the APE1 knockdown background (Figure 4C), which suggested that acetylation modification of APE1 may also be involved in melphalan resistance.

### MDR1 expression is reduced in APE1 deficient MM cells

**Figure 6:**
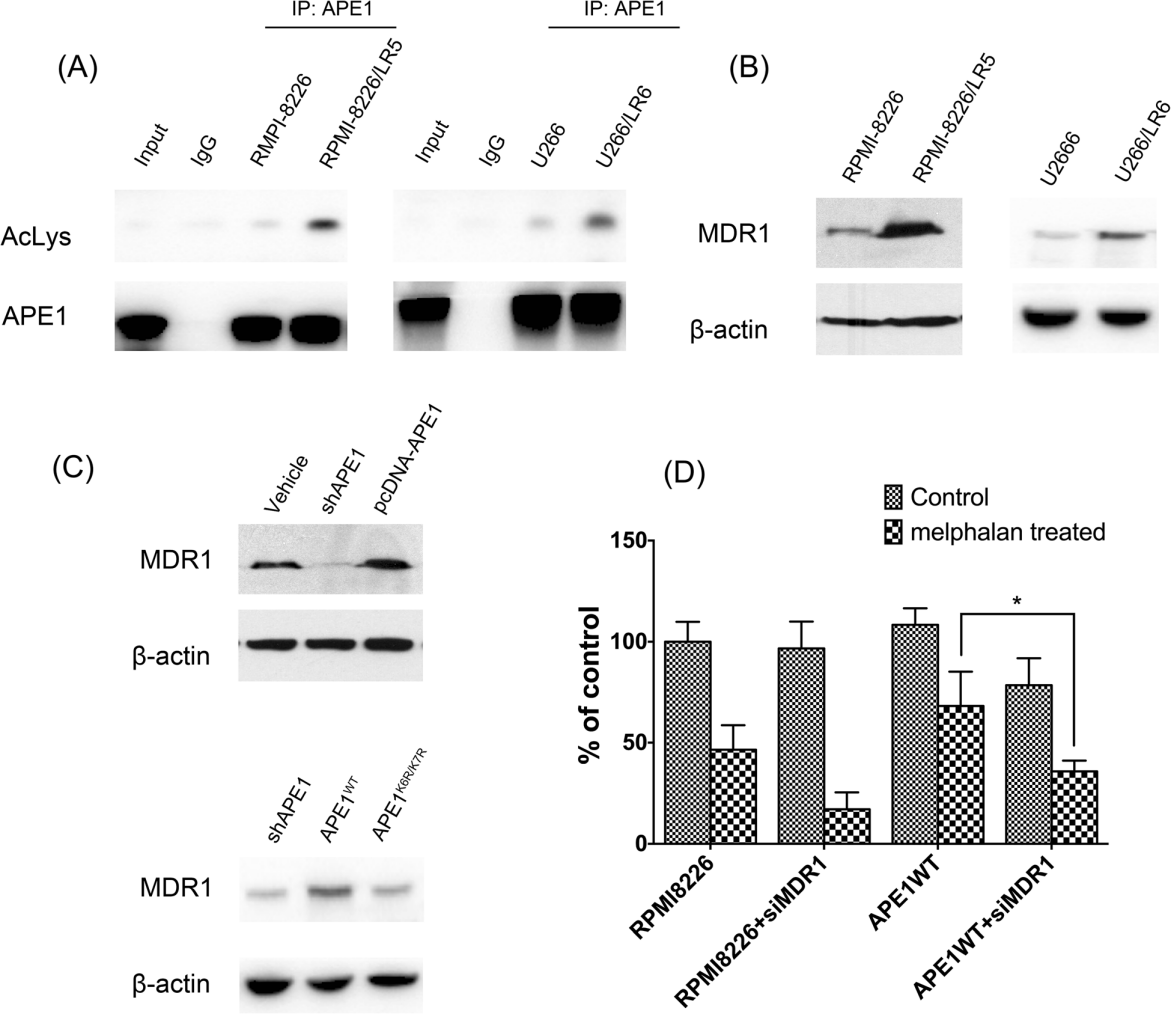
**MDR1 expression is aberrant in APE1 acetylation site mutant expressing MM cells. (A)** At 2 hours after melphalan treatment, APE1 was enriched by pulldown and blotted with anti-lysine acetylation antibody. **(B)** The Western blot showed expression levels of MDR1 in RPMI-8226/LR5, U266/LR6 and their parental cell lines RPMI-8226 and U266. The representative blots showed that melphalan resistant cells have higher expression levels of MDR1. **(C)** MDR1 protein expression was detected by Western blot at 48 hours after APE1 siRNA or pcDNA-APE1 was transfected into RPMI-8226 cells. MDR1 expression was downregulated after knockdown of APE1 in RPMI-8226 cells, and MDR1 was upregulated after overexpression of APE1. In addition, APE1 ^WT^ or APE1 ^K6R/K7R^ was transfected 24 hours after APE1 shRNA infection. 48 hours later, MDR1 levels were detected by Western blot. **(D)** MDR1 siRNA was applied to the pcDNA-APE1 transfected RPMI-8226 cells at 48 hours post 15 μM melphalan treatment, and cell viability was measured by a CCK-8 kit. The results were from three independent experiments.

We then analyzed the possible mechanism of APE1 acetylation-mediated melphalan resistance in MM cells. Firstly, we detected the acetylation level of APE1 in melphalan resistant MM cell lines and their wildtype counterparts. As shown in Figure 6A, APE1 acetylation levels could be detected when APE1 was enriched by immunoprecipitation. After normalization with pan APE1, its acetylation level increased in the melphalan-resistant MM cells RPMI-8226/LR5 and U266/LR6 in response to melphalan treatment. Due to the importance of MDR1 in drug resistance, we then measured differences in the expression of MDR1 between RPMI-8226-LR5 and its parental cell line. MDR1 is constitutively expressed to a higher level in melphalan-resistant MM cells as shown in Figure 6A. Since a previous study [18] reported that APE1 plays a critical role in regulation of MDR1 expression through a novel acetylation modification, we then postulated that APE1 could be involved in melphalan resistance in MM cells by regulating MDR1 expression. First we observed that APE1 knockdown and overexpression in the RPMI-8226 cells manipulated the MDR1 protein expression level (Figure 6B). We then tested if the melphalan resistance induced by APE1 overexpression could be negated by knocking down MDR1 expression. APE1 wildtype cDNA expression vector and a vector only control were transfected in parallel into RPMI-8226 cells. Both of these transfected cell lines were then transfected with siRNA specifically against Mdr1. Different groups of cells were then challenged with melphalan. Forty-eight hours later, the cell viability was tested by CCK-8 assay. The results indicated that APE1 overexpression enhanced melphalan resistance in RPMI-8226 cells, and siMDR1 sensitized RPMI-8226 to melphalan. However, when siMDR1 was combined with pcDNA-APE1, siMDR1 reduced cell survival after melphalan treatment. These results suggested APE1 benefits cell survival after melphalan treatment specifically through an MDR1-dependent mechanism.

## Discussion and conclusion

Despite the adverse side effects caused by alkylating agents on bone marrow and other normal tissues, melphalan remains one of the most commonly prescribed chemotherapies in MM patients. As the main treatment regimen of MM, melphalan greatly affects the outcome of overall treatment through its therapeutic efficacy. However, a considerable variation of therapeutic response to melphalan is observed clinically. We previously found that melphalan cytotoxicity closely correlates to the expression level of the multifunctional gene APE1 [15]. Our present results indicate that APE1 has a higher expression level in the melphalan resistant cell line RPMI-8226/LR5 and that expression of APE1 is effectively induced by melphalan in a dose- and time-dependent manner. We further employed APE1 knockdown and overexpression vectors to exogenously manipulate the APE1 levels in RPMI-8226/LR5 and its parental cell line RMPI-8226 which affected melphalan cytotoxicity. These results further reinforced the critical regulatory role of APE1 in melphalan resistance in different MM cell line models in accordance with our previous observations. Subsequently, we determined which functions of APE1 were critical for melphalan resistance by taking advantage of developed melphalan-resistant MM cell lines and APE1 function-specific mutant expression vectors. By comparing the different capacities of restoring resistance to APE1-knockdown RPMI-8226 cells by three APE1 functional mutants, the DNA repair activity and the intact acetylation residues K6 and K7 were shown to play critical roles in the development of melphalan resistance of MM cells. Then we performed mechanistic studies of DNA repair activity and acetylation modification of APE1 and the impact on melphalan cytotoxicity. Altogether our results identify the most important functions of APE1 in melphalan resistance of MM cells and shed light on future therapeutic strategies targeting specific APE1 functions by small molecule inhibitors.

As predicted, the DNA repair activity of APE1 plays the most important part in melphalan resistance. Melphalan, as a typical alkylation agent, exerts its cancer cell killing effect through DNA damage. Although the most cytotoxic lesion caused by melphalan is considered to be the interstrand crosslinks, the majority of DNA lesions are N^7^G monoadducts (~38%) and N^3^A monoadducts (~20%) [25] which are also potentially lethal by blocking DNA replication. DNA base alkylation and oxidative lesions are mainly repaired by BER, so it is plausible that the abasic site endonuclease activity of APE1 functions as a major mechanism in acquired melphalan resistance. Although the various single base lesions require different lesion-specific glycosylases, APE1 is common to BER and incises the backbone of the DNA strand and facilitates gap filling by DNA Polβ. Therefore, the activity of APE1, essential for BER, determines the repair capacity of mono methylation induced by alkylation agents including MMS [14] and melphalan. In addition, DNA repair activity is associated with the sensitivity of different agents targeting DNA which mainly cause different types of lesions including cisplatin [13], 5-FU [26], bleomycin, and ionizing radiation (IR) [27]. Although the detailed mechanisms of the repair activity of APE1 in drug resistance remain unclear, these observations imply a more important role of APE1 in promoting cell survival though a DNA repair-dependent mechanism.

We recently observed that APE1 was highly expressed in bone marrow stromal cells (BMSCs) in MM patients compared to the normal donors. This study provided a plausible explanation to the drug-resistance of MM by APE1 in that APE1 regulates cytokines, including IL-6 and IL-8, produced in BMSCs through redox regulation of NF-κB and AP-1 [28]. This study merely focused on the role of the microenvironment of MM. However, our present results indicate that the redox activity of APE1 is not involved in acquired melphalan resistance as we expected. We actually observed the reduction of IL-6/8 mRNA in redox activity deficient cells (data not shown), and the reduction of IL-6/8 expression has a minor impact on cell survival after melphalan treatment. The paracrine agents from the BMSCs in the microenvironment of the bone marrow are the major source of cytokines and growth factors for MM cell survival; hence, it is probable that the autocrine cytokines from MM cells have little effect on drug resistance.

Interestingly, we found that APE1 regulates the sensitivity of MM cells to melphalan by affecting MDR1 expression. This MDR1 regulatory role of APE1 is exclusively dependent on the integrity of acetylation sites at K6/K7 as reported previously [18]. A previous study indicated that the MDR1 expression level was associated with low intracellular accumulation and low cytotoxicity of melphalan in different hematopoietic cancer cell lines, including seven MM cell lines [29]. In accord with our study, the MDR1 inhibitor successfully reversed melphalan resistance in MDR1 overexpressed HL-60 cells. However, the regulatory role of APE1 in melphalan sensitivity occurs only partially through MDR1 expression. As shown in Figure 6, knockdown of MDR1 in APE1-overexpressed RPMI-8226 cells only partially restores sensitivity to melphalan compared to the MDR1 knockdown in RPMI-8226 cells.

In this present study we are the first to identify, to our knowledge, that the APE1 DNA repair function, together with acetylation modification, plays the most important role in melphalan resistance. Although we demonstrated the critical function of APE1 in melphalan resistance through cell models in this current project, the detailed molecular mechanisms in intrinsic resistance to melphalan are still unknown. Since different mechanisms may be involved in intrinsic and acquired melphalan resistance, more work needs to be done using different cell models to determine the different functions of APE1 in intrinsic and acquired melphalan resistance.

## Abbreviations

ABC: ATP-binding cassette; APE1: Apurinic/apyrimidinic endonuclease; BER: Base excision repair; BMSCs: Bone marrow stromal cells; CCK-8: Cell counting kit-8; EMSA: Electrophoretic mobility shift assay; IR: Ionizing radiation; MDR1: Multidrug resistance protein 1; MM: Multiple myeloma.

## Competing interests

The authors declare they have no conflict of interests pending.

## Authors’ contributions

JX, participated in design of the experiments, data analysis and manuscript writing, provied the funding. LZ, carried out the cell biologic experiments and Western blot, participated in manuscript writing. ML, carried out the molecular biologic experiments, participated in data analysis and manuscript writing. JD, carried out the comet assay. LZ, carried out the cell biologic experiments. SY, carried out the Co-IP. LZ, carried out the AP incision assay. ZL, participated in experimental coordination. GW, participated in data analysis. DW, participated in design of the experiments, data analysis and helped to draft the manuscript. All authors read and approved the final manuscript.

## Pre-publication history

The pre-publication history for this paper can be accessed here:

http://www.biomedcentral.com/1471-2407/14/11/prepub
